# Role of GIRK2 channels in morphine-induced metabolite changes in the rostral ventromedial medulla^☆^

**DOI:** 10.1016/j.mri.2026.110668

**Published:** 2026-03-20

**Authors:** Ozra Dehkordi, Stephen Lin, Safia F. Mohamud, Martha Davila-Garcia, Richard M. Millis, Paul C. Wang

**Affiliations:** a Department of Neurology, Howard University Hospital, Washington, D.C. 20060, United States; b Department of Radiology, Howard University Hospital, Washington, D.C. 20060, United States; c Department of Pharmacology, Howard University College of Medicine, Washington D.C. 20059, United States; d Department of Physiology, American University of Antigua College of Medicine, Antigua and Barbuda; e Department of Physics, Fu Jen Catholic University, Taipei, Taiwan

**Keywords:** Rostral ventral medulla, Morphine, GIRK^+^/^−^ channels, ^1^H NMR

## Abstract

**Background:**

The rostral ventromedial medulla (RVM) is a brainstem structure that integrates descending modulatory signaling and contains neurons highly responsive to opioid receptor activation. Despite the well-established effects of opioids in the RVM, the neurochemical adaptations following sustained morphine exposure remain poorly understood. In particular, the contribution of G-protein–coupled inwardly rectifying potassium type 2 (GIRK2) channels, key mediators of opioid receptor–dependent antinociception has not been fully characterized. We hypothesized that GIRK2 channels are essential for morphine-induced metabolic alterations in the RVM.

**Methods:**

In vivo proton nuclear magnetic resonance spectroscopy (^1^H NMR) was used to examine metabolite responses to prolonged morphine exposure. Metabolite profiles were compared between wild-type and GIRK2 heterozygous mutant (GIRK2^+^/^−^) mice before and after four days of subcutaneous implantation with placebo or morphine pellets.

**Results:**

In wild-type mice, morphine exposure significantly increased levels of phosphocreatine, total creatine, glutamine, glutathione, taurine, and glycerophosphocholine plus phosphocholine (GPC + PCh), while decreasing *N*-acetylaspartate (NAA). These changes suggest enhanced energy storage, activation of antioxidant pathways, increased membrane turnover, and alterations in neuronal integrity and excitatory neurotransmission. In contrast, GIRK2^+^/^−^ mice exhibited attenuated or opposite responses to morphine, characterized by elevated glutamate and reductions in glutamine, GPC + PCh, and total creatine, with no change in NAA. These differential responses indicate that GIRK2 channels influence neurochemical adaptations to morphine in the RVM.

**Conclusion:**

These findings identify the GIRK2 channel as an important modulator of morphine-induced metabolic changes in the RVM. The observed neurochemical alterations likely reflect adaptive responses to sustained opioid exposure.

## 1. Introduction

The rostral ventromedial medulla (RVM) is a central component of descending brainstem circuits involved in pain modulation, integrating signals from supraspinal structures to regulate spinal dorsal horn processing [1]. Within the RVM, distinct neuron classes have been identified: ON-cells, which facilitate nociceptive transmission; OFF-cells, which inhibit it; and NEUTRAL-cells, which contribute to autonomic regulation, including thermogenesis, cardiovascular control, and respiration [2–6]. The dynamic relationship between ON- and OFF-cell activity is essential for maintaining physiological balance in pain perception and modulation, and this balance is an important target of pharmacological intervention.

Opioids exert profound effects within the RVM by modulating the excitability of distinct neuronal populations. Systemic administration of morphine suppresses ON-cell activity and activates OFF-cells, thereby reducing nociceptive transmission [7,8]. The RVM expresses multiple opioid receptor subtypes, including μ-, κ-, δ-, and Nociceptin/Orphanin FQ receptor, with μ-opioid receptors particularly enriched on ON-cells [1,7,9]. Upon opioid binding, μ-opioid receptors activate Gi/o proteins, which dissociate into Gα and Gβγ subunits [10]. The released Gβγ subunits directly activate G-protein–gated inwardly rectifying potassium (GIRK/Kir3) channels, most prominently those containing the GIRK2 subunit, thereby promoting potassium efflux and membrane hyperpolarization [11]. This hyperpolarization decreases neuronal excitability and reduces the probability of action potential generation [10,11]. Concurrently, μ-opioid receptor signaling inhibits adenylyl cyclase, lowering intracellular cAMP, and suppresses presynaptic calcium influx, further diminishing neurotransmitter release [12]. Collectively, these mechanisms attenuate neuronal firing and synaptic transmission [13]. The critical role of GIRK2 in opioid signaling is highlighted by studies in weaver mutant mice, which carry a loss-of function mutation in the GIRK2 gene and exhibit attenuated analgesic responses to morphine, κ-opioid receptor agonists, and ethanol [12,13].

Despite extensive electrophysiological studies, the absence of molecular markers has limited detailed characterization of ON- and OFF-cells, leaving many neurotransmitters, receptors, and signaling pathways incompletely understood. Positron emission tomography (PET) using μ-opioid receptor tracers provides in vivo measures of receptor availability and activation, offering valuable insight into receptor binding and occupancy [14]. However, PET primarily reflects receptor-level pharmacodynamics rather than downstream neurochemical changes. In contrast, in vivo proton nuclear magnetic resonance (^1^H NMR) spectroscopy enables non-invasive quantification of metabolites such as phosphocreatine, glutamate, and glutathione, which serve as indicators of cellular energy metabolism, neurotransmitter cycling, and oxidative homeostasis [15,16]. This metabolic perspective complements receptor-based imaging by revealing biochemical adaptations that occur with sustained opioid exposure, thereby offering a more integrated understanding of opioid action beyond receptor engagement alone. Accordingly, in the present study we employed in vivo ^1^H NMR spectroscopy to characterize RVM metabolite profiles in wild-type and GIRK2 heterozygous mice subjected to continuous morphine or placebo administration. Building on prior findings of morphine-induced metabolic changes in other opioid-sensitive brain regions [15,16], and given that the RVM is a key opioid-responsive region involved in pain modulation we conducted an exploratory investigation of morphine-induced metabolic alterations while testing the specific hypothesis that GIRK2 channels mediate these effects in the RVM. We predicted that morphine exposure would alter metabolites related to energy metabolism (phosphocreatine, creatine), neurotransmission (glutamate, glutamine), antioxidant defense (glutathione, taurine), neuronal integrity (*N*-acetylaspartate), and membrane turnover (glycerophosphocholine + phosphocholine), with these effects differing markedly between GIRK2^+^/^−^ and wild-type mice.

## 2. Methods

### 2.1. Animals

Wild-type and GIRK2 heterozygous (GIRK2+/−) mice (male and female, 5–6 weeks old) were obtained from the Jackson Laboratory (Bar Harbor, ME, USA). Mice aged 5–6 weeks were selected to model adolescence, a developmental stage characterized by heightened neuroplasticity and increased vulnerability to opioid exposure. Opioid administration during adolescence has been shown to induce long-lasting neurobiological alterations, making this developmental window particularly relevant for examining opioid-related changes in brain metabolites [17]. The GIRK2+/− mice were generated via a double Custom Embryo Cryorecovery of B6CBACa Aw-J/A-Kcnj6wv/J (weaver, Stock #000247) mice. The resulting mice were crossed with B6CBAF1/J (Stock #100011) mice to establish a stable breeding colony consisting of wild-type and heterozygous GIRK2+/− mixed-sex mice, specifically for this study.

A total of 32 mice, 16 wild-type and 16 GIRK2+/− mice, were randomly divided into two treatment groups (*n* = 8 per group) to receive either morphine or placebo pellets. All procedures, including anesthesia and surgery, were approved by the Institutional Animal Care and Use Committee (IACUC) of Howard University. All efforts were made to minimize the number of animals used and their suffering. To reduce the nonspecific effects of handling and experimental environment, animals were handled and exposed to the same environment as those used during the actual experiment for a total of 3 days (Handling Day 1–3). On Day 4, animals were anesthetized (isoflurane in 0.8 L/min oxygen at 2–3% for induction, 1–2% for maintenance) and underwent basal (pre-exposure) ^1^H NMR. On Day 5, mice underwent subcutaneous implantation of morphine pellets (75 mg) or placebo pellets (NIDA, Bethesda, MD, USA) according to previously described protocols [18,19]. The mice were anesthetized with 2.5% isoflurane before shaving their hair from the base of the neck. The skin was cleansed with 10% povidone-iodine and rinsed with 70% ethanol before making a 1- cm incision at the cleansed area. The subcutaneous space was opened, and the pellets were inserted in the space. The site was closed with GLUture topical tissue adhesive (Zoetic Inc., Kalamazoo, MI). The animals were allowed to recover in their home cages. Since the procedure involved only a small subcutaneous incision under isoflurane anesthesia, it was considered minimally invasive. To avoid introducing variables that could interfere with the interpretation of morphine’s effects on the RVM, no additional postoperative analgesia was administered. Four days after implantation of pellets, the animals were anesthetized and underwent a second ^1^H MRS, and immediately thereafter, animals were euthanized.

### 2.2. ^1^H NMR spectroscopy

An animal monitoring unit (SA Instruments, Stony Brook, NY) was used to monitor respiration during imaging and magnetic resonance spectroscopy acquisition. Depth of anesthesia was assessed throughout the study by monitoring the respiration rate, with target respiration range 40–50/min during scanning. A water circulation system set to 32 °C was used to maintain body temperature within the thermoneutral range for mice (30–32 °C), minimizing temperature-driven metabolic variability during ^1^H NMR measurements [20]. A 9.4 T Bruker AVANCE 89 mm vertical bore MRI machine (Bruker, Billerica, MA) was used with a 25 mm RF volume coil. A set of three orthogonal T1-weighted 2D Rapid Acquisition with Refocused Echoes (RARE) fast spin echo (TE = 8.4 ms, TR = 800 ms, RARE Factor = 4, NA = 2, FA = 90°/180°, FOV = 2 × 2 cm, MTX = 256 × 192, slice thickness = 1 mm) and an axial T2-weighted 2D RARE (TEeff = 37 ms, TR = 2500 ms, RARE Factor = 8, NA = 8, FA = 90°/180°, FOV = 1.28 × 1.92 cm, MTX = 192 × 192, slice thickness = 0.5 mm) pilot images were acquired in an orientation matching a mouse stereotactic brain atlas [21] to identify key landmarks for positioning the region of interest (ROI) for spectroscopy. A B_0_ fieldmap-based localized shimming was applied over the ROI, followed by iterative first order localized shimming, nominally achieving <15 Hz full-width half-max water peak. Localized ^1^H NMR was acquired with a point resolved spectroscopy (PRESS) sequence (TE = 15 ms, TR = 2.5 s, 1024 averages) with variable power and optimized relaxation (VAPOR) delays for water suppression from RVM region; an unsuppressed water reference was also acquired as part of his sequence from the same ROI using PRESS (TE = 15 ms, TR = 2.5 s, 1 average) and saved separately. It was estimated that 2048–4096 averages would be necessary for acceptable signal-to-noise ratio based on the ROI. However, because long acquisition times increase the risk of anesthesia complications, a set of 4 PRESS sequences with 1024 averages was acquired one after the other, which would be combined during data processing. This approach allowed prematurely ending the study if the animal no longer tolerated the anesthesia while preserving useful data and allowing exclusion of individual scans containing motion or other artifacts.

The ROI for RVM was 2.0 × 0.7 × 1.2 mm^3^. ROI placement was referenced to the Paxinos and Franklin mouse brain atlas [20] but guided primarily by clearly identifiable anatomical landmarks visible on MRI pilot images including the pyramids, cerebellar white matter, and the fourth ventricle. These structures provide robust MRI boundaries, and stereotaxic localization relies on stable cranial landmarks (bregma, lambda, midline), minimizing sensitivity to age-related differences in brain size [22]. The ROI (bregma −5.41 mm to −6.59 mm) in the axial view was placed on the midline approximately 0.5 mm above the pyramids, using cerebellar white matter and ventricle morphology as reference markers.

### 2.3. Data processing and statistical analysis

Each of the 4 spectra from each mouse and time point was examined and individually processed with LCModel [23]. LCModel uses a linear combination of model spectra acquired from in vitro solutions to fit the acquired spectra. A basis set of these model spectra, matching the field strength and acquisition parameters of the study (TE = 15 ms), was generated and provided by Dr. Provencher of LCModel Inc. This basis set includes L-alanine (Ala), aspartate (Asp), creatine (Cr), phosphocreatine (PCr), γ-aminobutyric acid (GABA), glucose (Glc), glutamine (Gln), glutamate (Glu), glycerophosphocholine (GPC), phosphocholine (PCh), glutathione (GSH), myo-inositol (Ins), lactate (Lac), N- acetylaspartate (NAA), *N*-acetylaspartylglutamate (NAAG), scyllo-inositol (Syllo-Ins), and taurine (Tau), along with simulated lipids and macromolecule compounds. Using an unsuppressed water reference, LCModel provides metabolite concentrations along with their associated Cramér-Rao Lower Bounds (CRLB), expressed as percentages (%SD). The CRLB represents the uncertainty in the concentration estimate, with lower values indicating more reliable quantification; metabolite concentrations with a %SD below 20% were considered reliable and included in the final statistical analyses, while higher %SD values indicate greater uncertainty. For initial quality assessment, spectra with most or all metabolite concentration %SDs above 50% were excluded. Of the 236 spectra acquired, 11 were excluded for failing to meet this quality threshold, possibly due to motion from the animal momentarily becoming under- or over-anesthetized during the long duration of anesthesia. The free induction decay (FID) of each spectra and their respective unsuppressed water reference were then pre-processed with LCModel’s preprocessor for referencing (frequency correction), phasing, and file type conversion, and then summed using a custom MATLAB (MathWorks, Natick, MA) script to generate a summed FID per animal per time point. The resulting summed FIDs were processed using LCModel to obtain the absolute concentrations of neurotransmitters and metabolites in the RVM.

Weighted means and standard deviations (SDs) of metabolite concentrations were calculated for each genotype, treatment group, and time point. Only metabolites with %SD < 20% across genotypes, treatment groups, and time points were included in the final analysis. Each animal contributed two weighted measurements per metabolite (Day 0 and Day 4). Because this yielded one value per subject per time point, and the sample size did not support a full factorial ANOVA, within-group changes were assessed using paired *t*-tests, and between-group differences were evaluated using independent t-tests. Because weighted means and their associated SDs were available for each metabolite at both time points, the change in concentration (Day 4 – Day 0) was computed for each metabolite, and the standard error of these change scores was derived directly from the SD of the weighted means, without requiring propagation of CRLB-based uncertainties. Statistical significance was defined as *P* < 0.05.

### 2.4. Intra-scan and inter-subject reproducibility

Intra-scan reproducibility was evaluated using the baseline metabolite concentrations for both wild-type and GIRK2+/− mice, where *n* = 30. Four consecutive baseline spectra were acquired for each animal. Each spectrum was processed independently with LCModel, and scans with CRLB values >50% for most metabolites were excluded (2 scans). Metabolites concentration estimates with CRLB %SD higher than 50% were excluded, resulting in 3 scans from GPC + PCh, 0 from NAA + NAAG, 1 from Cr + PCr and 1 from Glu + Gln being excluded. The mean (M) concentration, standard deviation (SD), and coefficient of variation (CV; calculated as SD/M × 100%) were determined for each metabolite for each animal. The CVs for each metabolite was then averaged across all animals. Lower CV values were interpreted as indicating greater intra-scan reproducibility of the metabolite measurements.

Inter-subject reproducibility of baseline metabolite concentrations was evaluated for both wild-type and GIRK2+/− mice across different scanning days. For each genotype, the mean (M) concentration, standard deviation (SD), and coefficient of variation (CV; calculated as SD/M × 100%) were determined for NAA + NAAG, GPC + PCh, Glu + Gln, and Cr + PCr. These metrics were computed separately for wild-type mice and GIRK2+/− mice, as well as for the combined cohort. Lower CV values were interpreted as indicating greater inter-scan reproducibility of the metabolite measurements.

## 3. Results

**Fig. 1.**
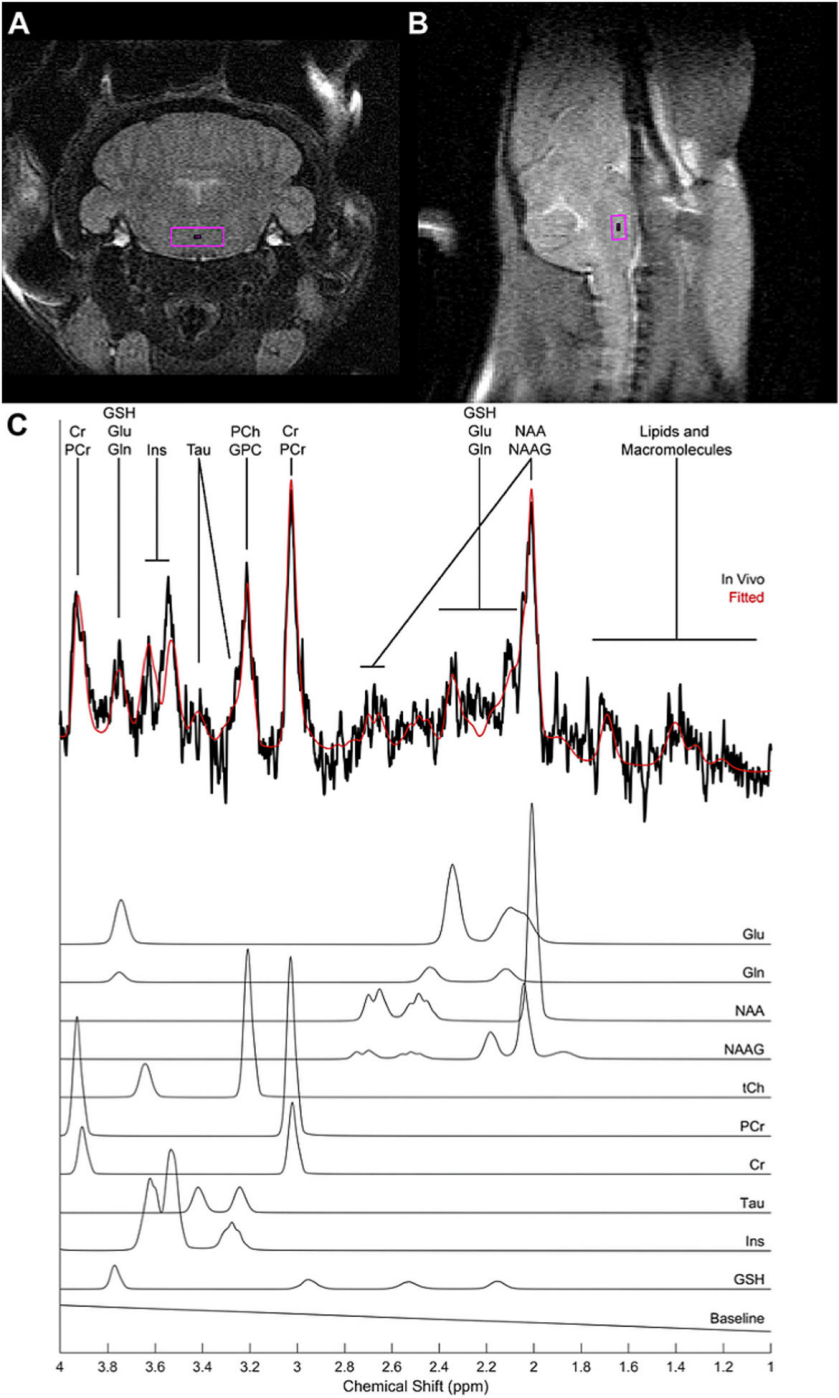
^1^H Nuclear magnetic resonance (^1^H NMR) spectroscopy at the rostral ventromedial medulla (RVM). Representative in vivo ^1^H NMR obtained from the RVM of a control mouse. Panels A and B: Axial and sagittal pilot images, respectively, highlighting the RVM region of interest (size 2.0 × 0.7 × 1.2 mm^3^). Panel C: Typical spectrum from the RVM region of interest in panels A and B with fitted spectrum from LCModel (red) and fitted metabolite subspectra. Absolute concentration of neurological biochemicals such as glutamate (Glu), glutamine (Gln), *N*-acetylaspartate (NAA), *N*-acetylaspartylglutamate (NAAG), phosphocholine (PCh), glycerophosphocholine (GPC), total creatine (tCr), taurine (Tau), inositol (Ins) and glutathione (GSH) can be identified in a naïve animal. (For interpretation of the references to colour in this figure legend, the reader is referred to the web version of this article.)

Fig. 1 presents a representative in vivo ^1^H NMR spectroscopy spectrum obtained from the RVM of a control mouse. Neurochemical profiles, comprised of multiple neurotransmitters and metabolites, were quantified using LCModel software. Complete numerical metabolite concentrations for Day 0 and Day 4 across all treatment groups are summarized in Supplemental Tables 1 and 2.

### 3.1. Comparison of baseline metabolite levels between wild-type and GIRK2^+^/^−^ mutant mice

**Fig. 2.**
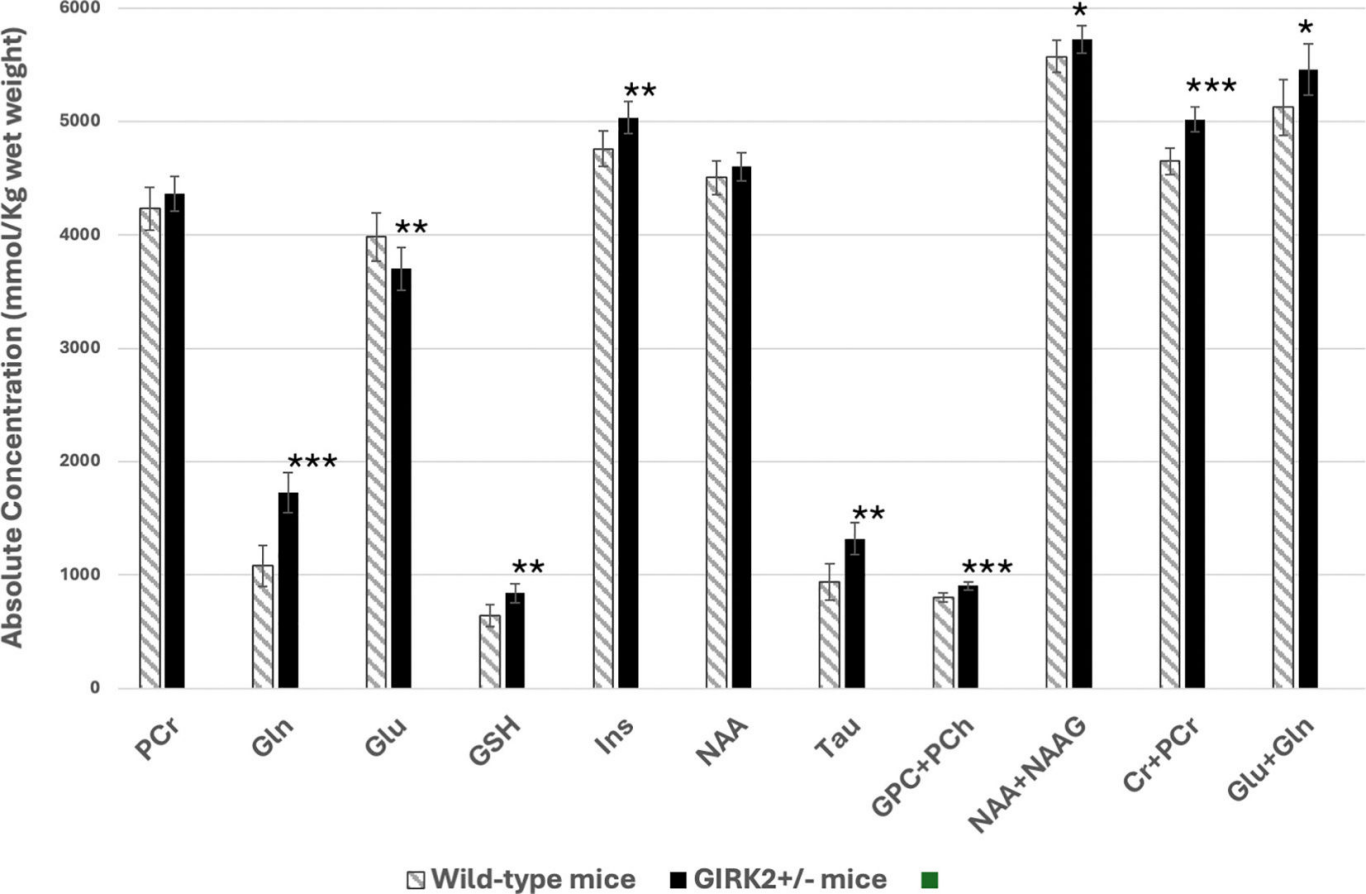
Baseline metabolite differences in the rostral ventromedial medulla (RVM) between wild-type and GIRK2^+^/^−^ mutant mice. Metabolite concentrations were quantified from the RVM on Day 0, prior to subcutaneous morphine administration. Data are presented as mean ± SD. Significance levels: *P < 0.05, **P < 0.01, ***P < 0.001.

At baseline (Day 0), the concentrations of several metabolites differed significantly between wild-type and GIRK2^+^/^−^ mice. Specifically, the levels of Gln, Glu, GSH, Ins, Tau, combined NAA + NAAG, GPC + PCh, Glu + Gln and Cr + PCr were significantly different in GIRK2^+^/^−^ mice compared to wild-type controls (Fig. 2). However, the concentrations of PCr and NAA did not differ significantly between wild-type and GIRK2^+^/^−^ mice.

### 3.2. Placebo-induced metabolite changes in wild-type and GIRK2^+^/^−^ mutant mice

**Fig. 3.**
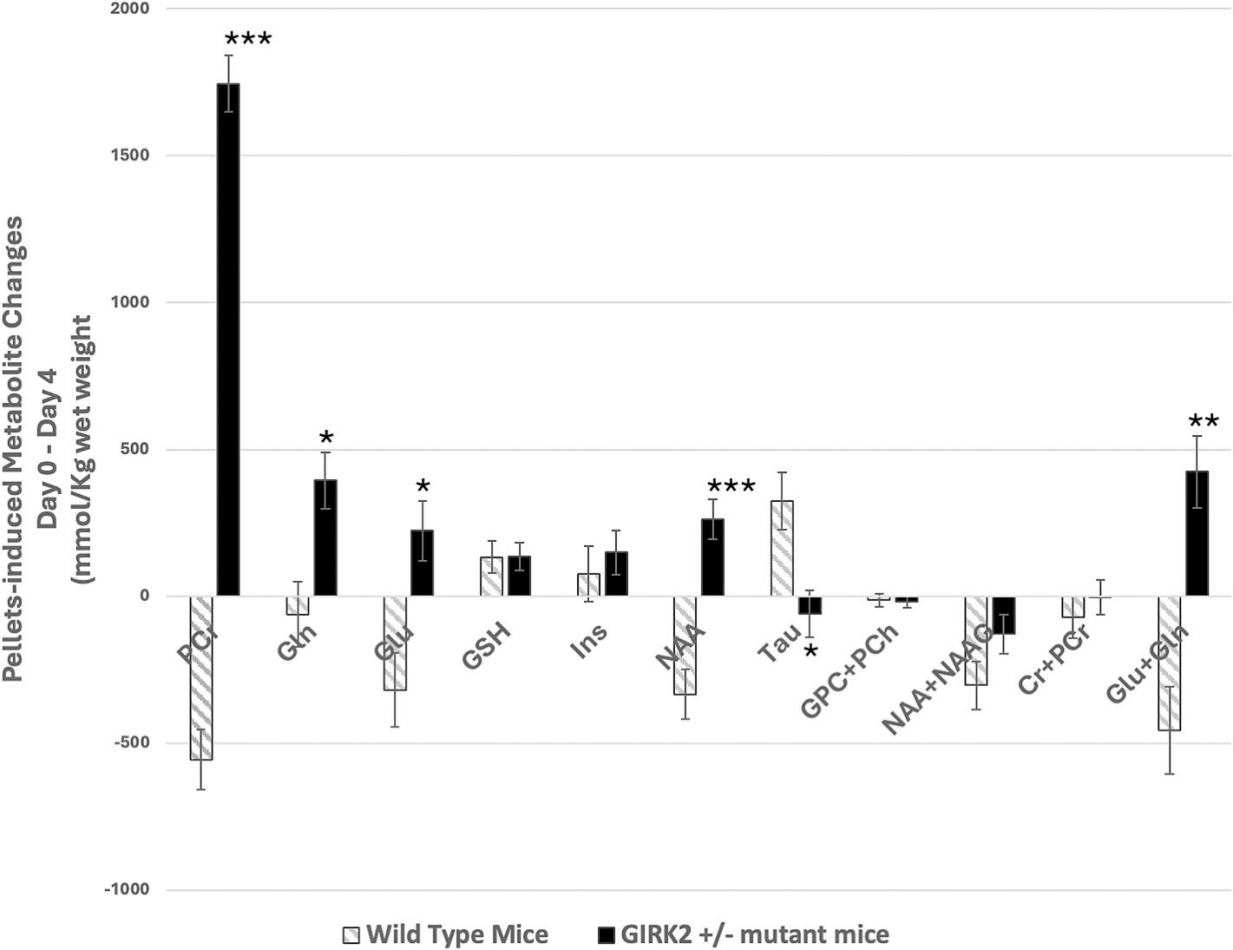
Comparison of placebo-induced metabolite changes at the rostral ventromedial medulla (RVM) of wild-type and GIRK2^+/−^ mutant mice. Changes in the concentrations of metabolites between Day 0 (no treatment) and Day 4 of placebo treated wild-type and GIRK2^+/−^ mutant mice. Data represents mean concentration differences for each metabolite (Day 0 - Day 4) ± SE. Significance levels: *P < 0.05, **P < 0.001, ***P < 0.001.

Comparison of placebo-induced metabolite changes in RVM between Day 0 and Day 4 revealed a significant difference between the wild-type and GIRK2+/− mutant mice (Fig. 3). In wild-type mice, Tau increased, while PCr, Gln, Glu, NAA, and the combined Glu + Gln pool decreased following placebo administration. In contrast, GIRK2^+^/^−^ mice exhibited an increase in PCr, Gln, Glu, NAA, and Glu + Gln, along with a decrease in Tau. Other metabolites remained unchanged in both genotypes.

### 3.3. Metabolite changes in the RVM of wild-type mice after morphine treatment

**Fig. 4.**
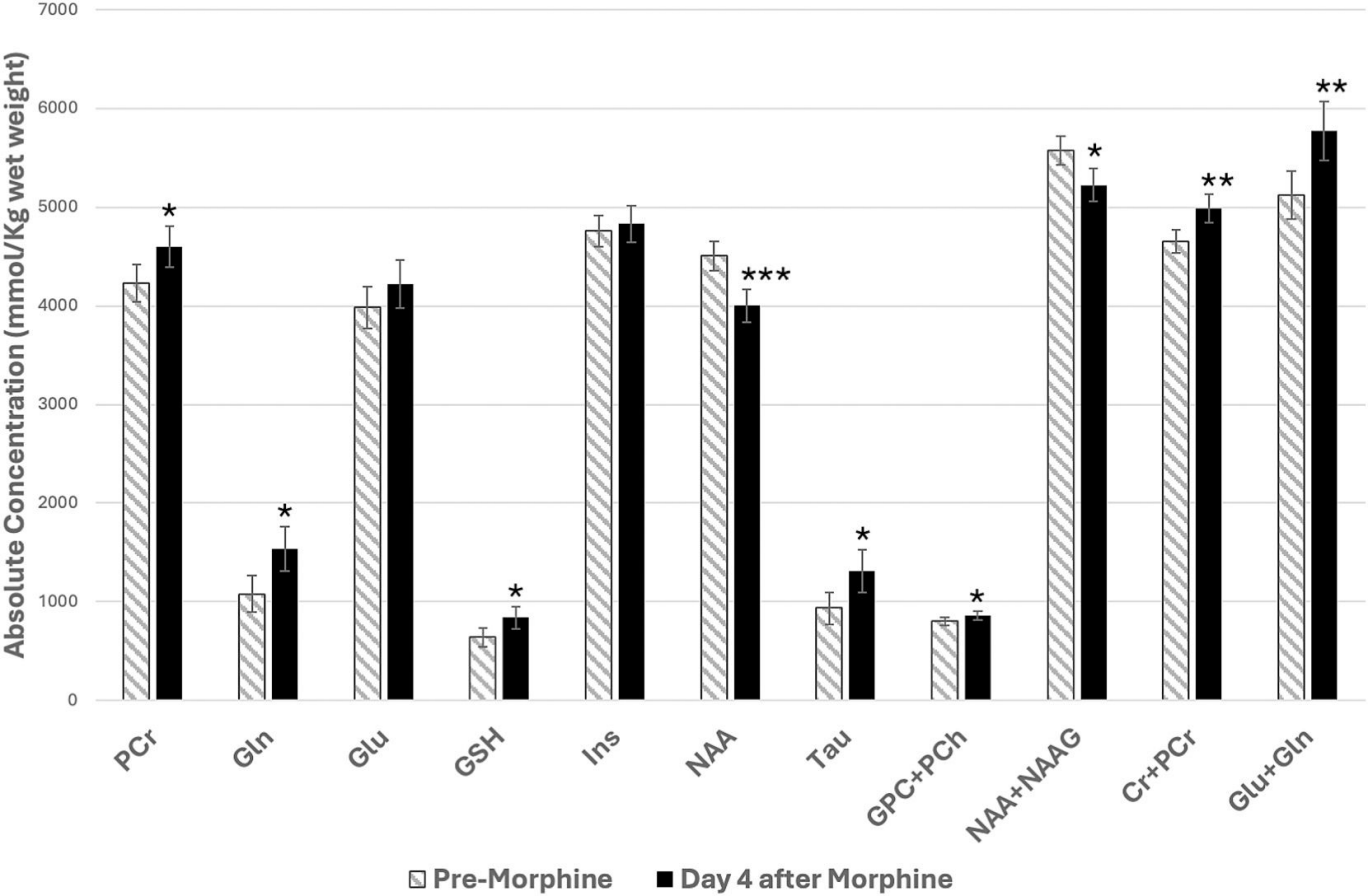
Morphine-induced metabolite changes at the rostral ventromedial medulla (RVM) of wild-type mice. Metabolite concentrations were quantified from the RVM of mice before (Day 0) and 4 days after subcutaneous administration of morphine. Data are presented as mean ± SD. Significance levels: *P < 0.05, **P < 0.01, ***P < 0.001.

**Fig. 5.**
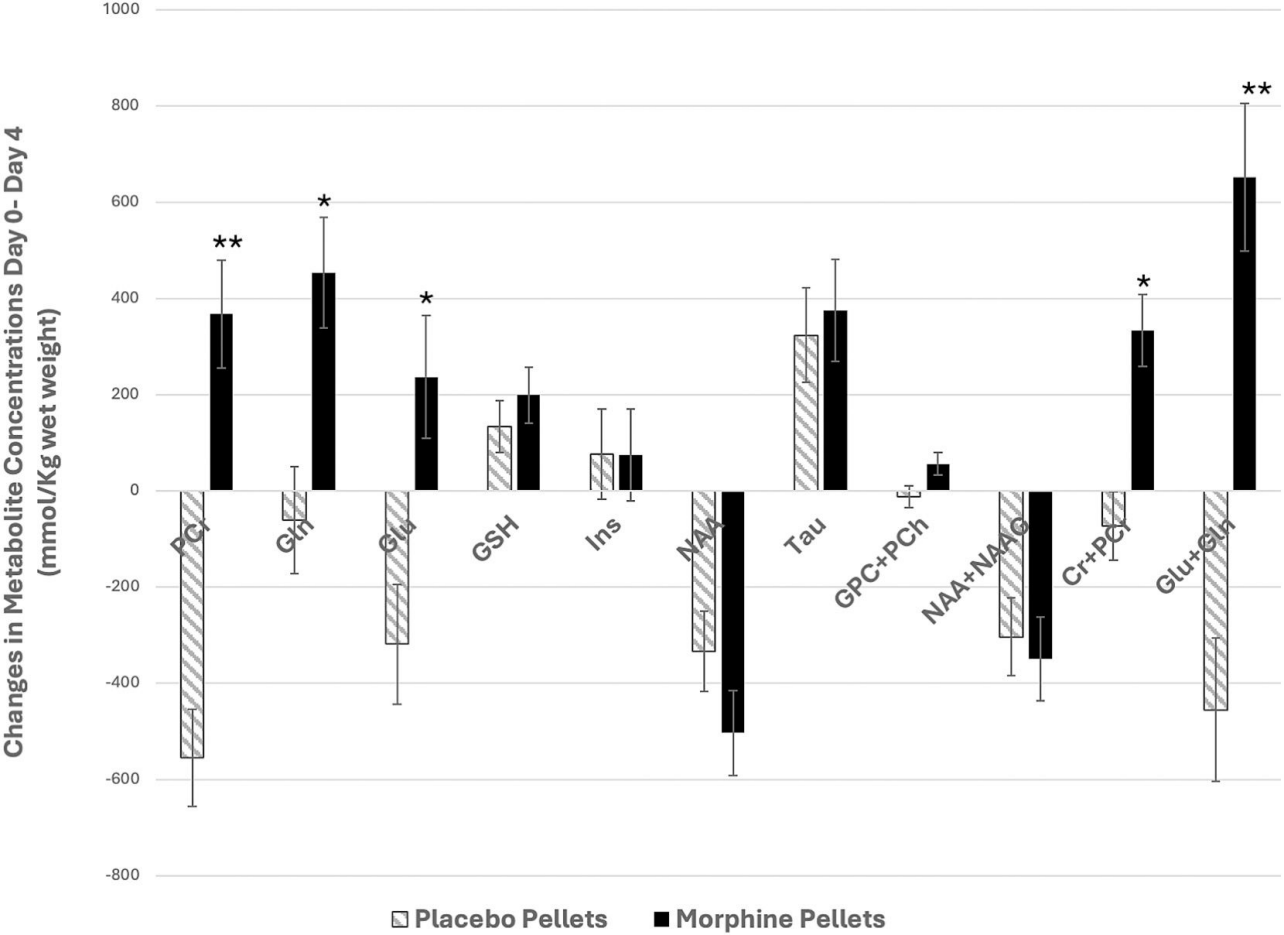
Comparison of placebo- and morphine-induced metabolite changes at the rostral ventromedial medulla (RVM) of wild-type mice. Changes in the concentrations of metabolites between Day 0 (no treatment) and Day 4 of placebo- and morphine-treated mice. Data represents mean concentration differences for each metabolite (day 0 - day 4) ± SE. Significance levels: *P < 0.05, **P < 0.01.

Subcutaneous administration of morphine produced significant changes in the concentrations of a number of metabolites in the RVM (Fig. 4). Concentrations of PCr, Gln, GSH, Tau, combined pool of GPC+ PCh, Cr + PCr, and Glu + Gln, exhibited significant increases. Conversely, NAA and NAA + NAAG concentrations were significantly reduced. However, Ins and Glu did not change significantly after morphine. Comparative analysis of the metabolite changes between Day 0 and Day 4 in placebo- and morphine-treated animals revealed significant differences in the directional changes in PCr, Gln, Glu, Glu + Gln, and Cr + PCr between the two groups. However, no statistically significant differences were observed in the concentrations of other measured metabolites between the two groups (Fig. 5).

### 3.4. Metabolite changes in the RVM of GIRK2 +/− mutant mice after morphine treatment

**Fig. 6.**
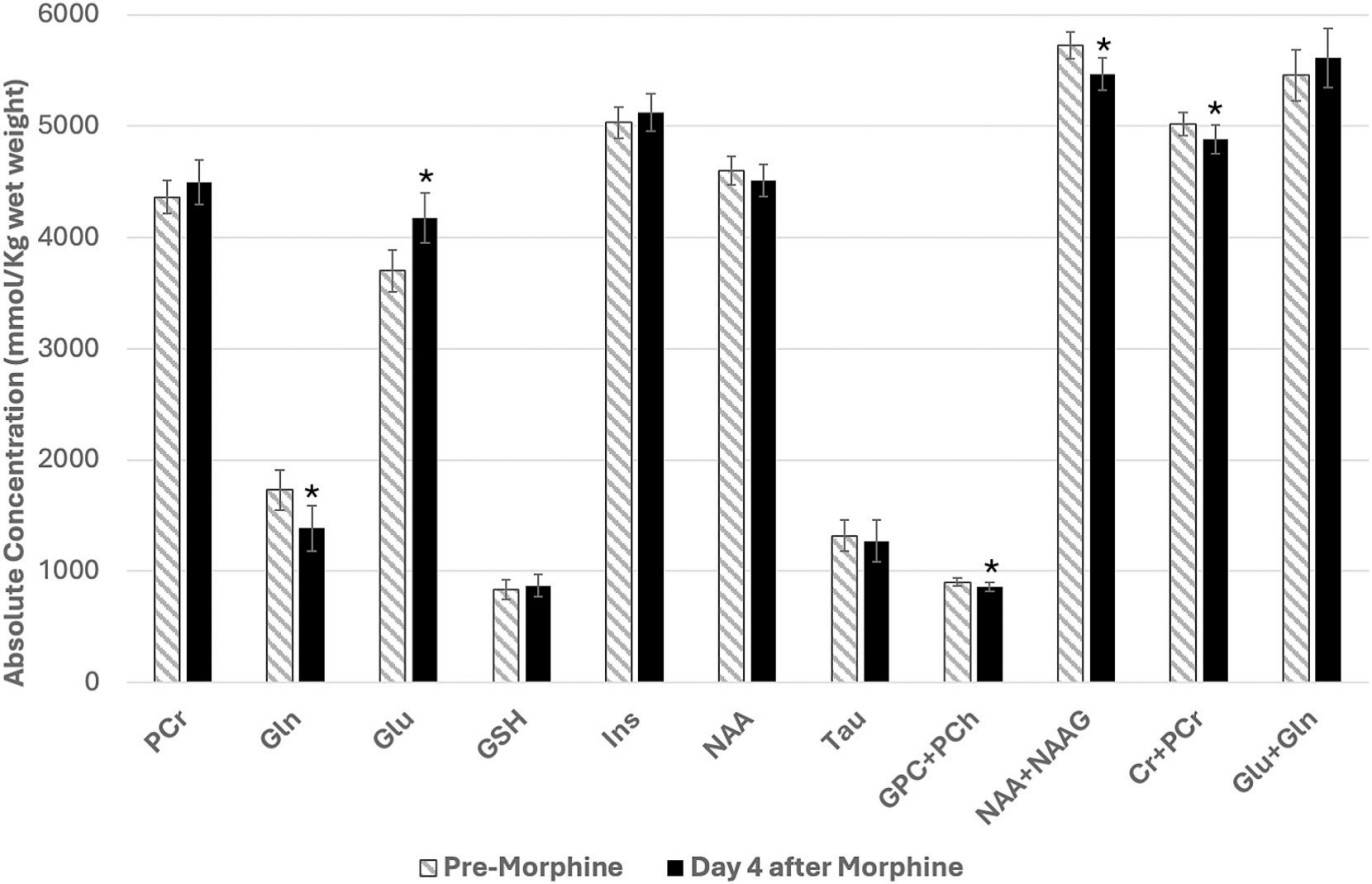
Morphine-induced metabolite changes at the rostral ventromedial medulla (RVM) of GIRK2^+^/^−^ mutant mice. Metabolite concentrations quantified from RVM of mice before (Day 0) and 4 days after subcutaneous administration of morphine. Data represent mean ± SD. Significance level: *P < 0.05.

**Fig. 7.**
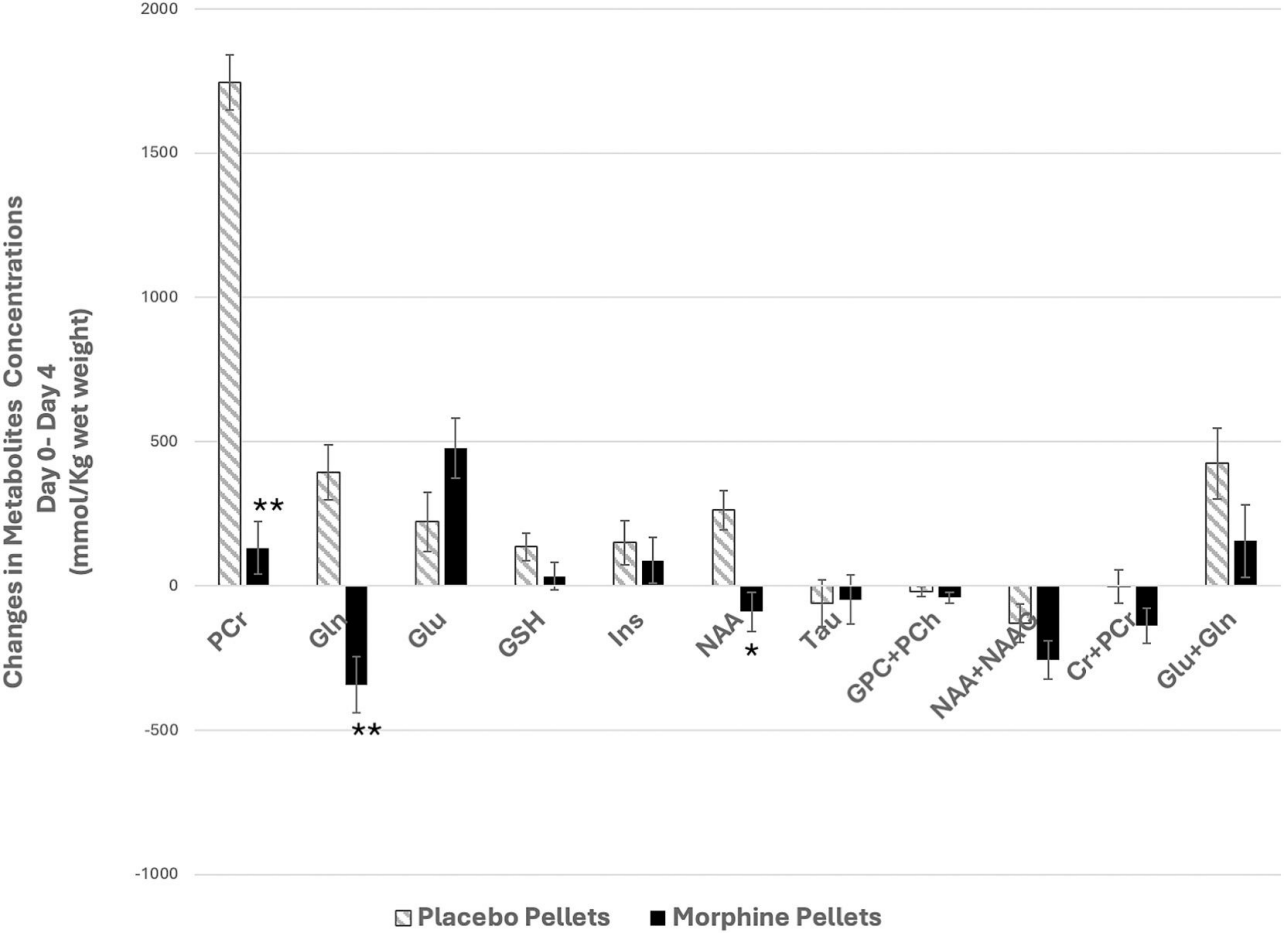
Comparison of placebo- and morphine-induced metabolite changes at the rostral ventromedial medulla (RVM) of GIRK2^+/−^ mutant mice. Changes in the concentrations of metabolites between Day 0 (no treatment) and Day 4 of placebo- and morphine-treated mice. Data represents mean concentration differences for each metabolite (Day 0 - Day 4) ± SE. Significance levels: *P < 0.05, **P < 0.001.

In the GIRK2+/− mutant mice, morphine also produced significant changes in the concentrations of several metabolites in the RVM (Fig. 6). Glu increased significantly after morphine. Gln, GPC + PCh, NAA + NAAG and Cr + PCr decreased but concentrations of other metabolites such as PCr, Ins, Tau, GSH, NAA and Glu + Gln did not change. Comparison of the changes in the concentrations of metabolites between Day 0 and Day 4 of the placebo and morphine treated GIRK2+/− mutant mice showed significant differences between the two groups with respect to either the direction and/or the magnitude of changes in PCr, Gln, and NAA (Fig. 7). However, concentrations of other metabolites including Glu, GSH, Ins, Tau, GPC + PCh, NAA + NAAG, Cr + PCr and Glu + Gln did not change between the two groups.

### 3.5. Comparison of morphine-induced metabolite changes in wild-type and GIRK2+/− mutant mice

**Fig. 8.**
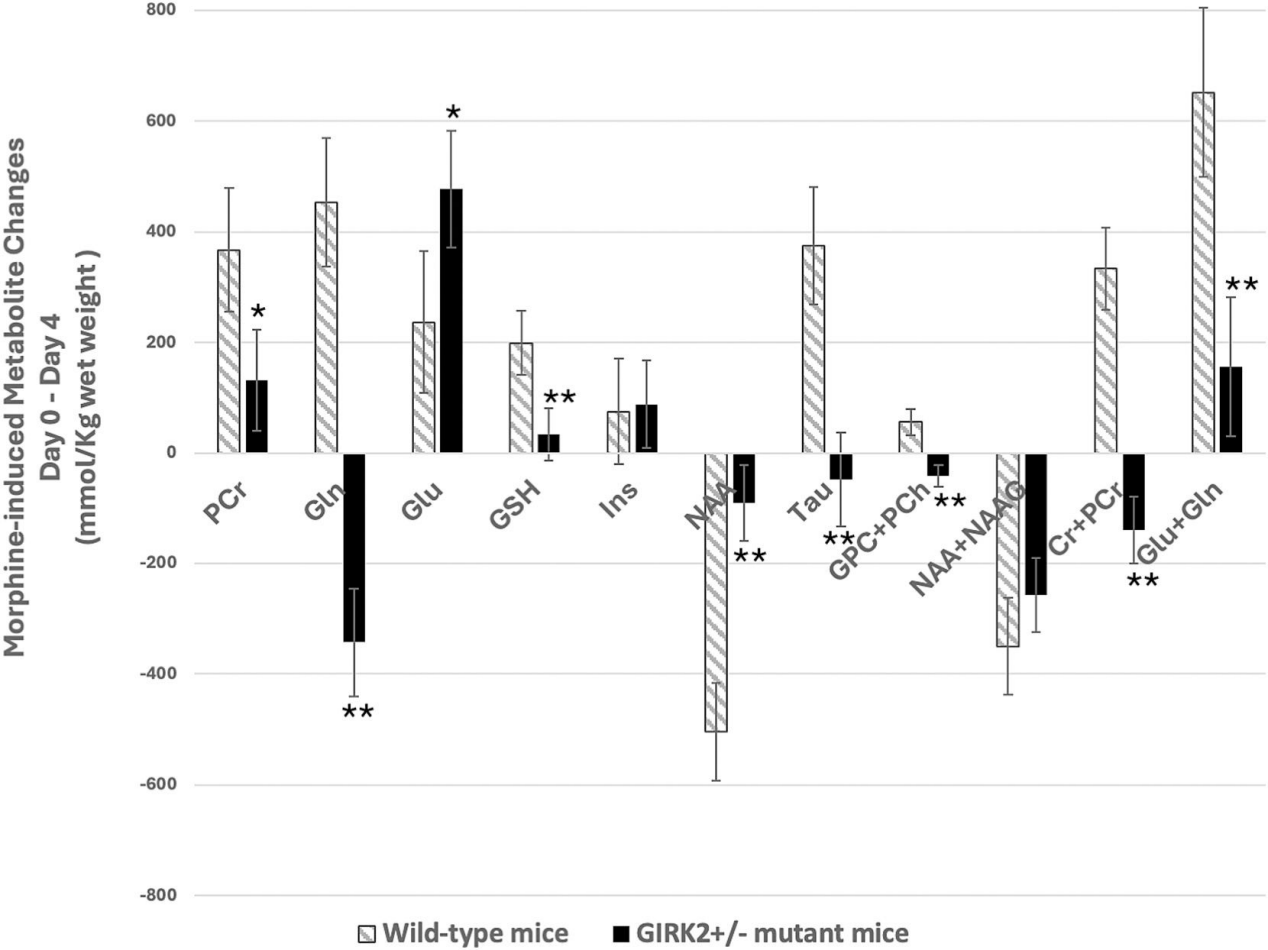
Comparison of morphine-induced metabolite changes at the rostral ventromedial medulla (RVM) of wild-type and GIRK2^+/−^ mutant mice. Changes in the concentrations of metabolites between Day 0 (no treatment) and Day 4 of morphine treated wild-type and GIRK2^+/−^ mutant mice. Data represents mean concentration differences for each metabolite (Day 0 - Day 4) ± SE. Significance levels: *P < 0.05, **P < 0.001.

Comparison of morphine induced metabolite changes in RVM between Day 0 and Day 4 of wild-type and GIRK2+/− mutant mice demonstrated a significant difference between the two groups with respect to either the magnitude and/or direction of changes in PCr, Gln, Glu, GSH, NAA, Tau, GPC + PCh, Cr + PCr and Glu + Gln. However, changes in the concentrations of Ins and NAA+ NAAG were not significantly different between the two groups (Fig. 8).

### 3.6. Intra-scan and inter-subject reproducibility

Intra-scan reproducibility analyses demonstrated stable metabolite quantification across baseline sessions. Mean CV% values for the major metabolites were generally low in both genotypes, indicating consistent measurement stability across repeated sessions. NAA + NAAG (12.4%), Cr + PCr (12.9%), and Glu + Gln (17.9%) exhibited mean CV% values below the commonly referenced reproducibility threshold of 20%, while GPC + PCh had a slightly higher mean CV% of 22.3%.

Inter-subject reproducibility analyses demonstrated stable metabolite quantification across baseline sessions in both the wild-type and the GIRK+/− mice. CV% values for the major metabolites were generally low in both genotypes, indicating consistent measurement stability across repeated sessions. All metabolites exhibited CV% values below the commonly referenced reproducibility threshold of 20%, with the exception of a single metabolite showing a CV of 20.8%. Reproducibility patterns were comparable between wild-type and GIRK+/− mice, supporting the reliability of the baseline metabolite estimates used in this longitudinal study. Detailed CV% values for each metabolite within each genotype and combined cohort are provided in Supplemental Tables 3 and 4.

## 4. Discussion

The present in vivo ^1^H MRS study in mice reveals that subcutaneous morphine administration leads to significant alterations in metabolite concentrations within the RVM of both wild-type and GIRK2^+^/^−^ mutant mice. Baseline metabolite levels differed significantly between GIRK2^+^/^−^ mutants and wild-type controls, indicating genotype-dependent metabolic patterns that are independent of morphine exposure. The observed baseline variations in metabolites involved in energy metabolism, neurotransmission, antioxidant activity, and choline-containing compounds suggest that GIRK2 channel dysfunction disrupts intrinsic neurochemical homeostasis in the RVM [10,11]. These baseline differences are consistent with previous evidence showing that GIRK2 channels influence neuronal excitability and neurotransmitter cycling [10–13]. Placebo pellet implantation also induced metabolic alterations in the RVM, likely due to surgical stress and local inflammatory responses. Interestingly, placebo produced opposite metabolic patterns in the two genotypes. The wild-type mice exhibited decreases in PCr, Glu, Gln, NAA, and Glu + Gln, consistent with increased energy utilization and neuronal activation under stress. Whereas GIRK2^+^/^−^ mice showed increases in these metabolites, suggesting a distinct stress adaptation mechanism potentially linked to impaired GIRK2-mediated inhibitory signaling and compensatory energy buffering. These findings suggest that GIRK2 channels may be modulators of neurochemical responses to stress, independent of opioid exposure.

Building on these baseline and stress-related differences, morphine administration elicited distinct, genotype-dependent metabolic responses, with both the magnitude and direction of changes differing substantially between wild-type and GIRK2^+^/^−^ mice. These differences suggest that GIRK2 channel dysfunction modulates the neurochemical impact of morphine within the RVM. However, it is important to emphasize that these findings reflect morphine-induced neurochemical modulation and should not be interpreted as direct evidence of behavioral analgesia, because behavioral assessments were not performed in this study.

Phosphocreatine (PCr), a high-energy phosphate donor critical for ATP synthesis and cellular energy buffering [24,25], was elevated following morphine administration in the wild-type mice (*P* = 0.007), whereas no such change was observed in GIRK2^+^/^−^ mutant mice. Elevated PCr has previously been associated with conditions involving reduced metabolic activity or altered energy homeostasis in the brain [24,25]. In the RVM, morphine’s suppression of neuronal excitability through μ-opioid receptor activation and subsequent GIRK channel opening [10,11] may plausibly decrease local energy consumption, thereby favoring relative PCr accumulation. Astrocytic contributions may also play a role, as glial regulation of creatine–phosphate shuttling is central to maintaining energy balance and supporting neurotransmitter cycling [26]. Taken together, the observed PCr increase in wild-type mice likely reflects a shift in cellular energy buffering and utilization under morphine-induced changes in neuronal and glial activity, rather than indicating analgesia per se. The absence of this metabolic shift in GIRK2^+^/^−^ mutants further suggests that intact GIRK2 signaling is necessary for morphine-induced modulation of energy metabolism within the RVM.

Prolonged morphine administration also significantly altered the glutamate-glutamine (Glu-Gln) cycle in both wild-type and GIRK2^+^/^−^ mutant mice. In wild-type mice, morphine increased levels of Gln as well as the combined pool of Glu + Gln. In contrast, in GIRK2^+^/^−^ mutant mice, morphine decreased Gln, increased Glu, and left the total Glu + Gln pool unchanged. These divergent responses suggest that GIRK2 channels influence morphine’s effects on glutamatergic neurotransmission, possibly by modulating astrocytic uptake or neuronal recycling mechanisms involved in the Glu-Gln cycle [26]. Further investigation is needed to elucidate the specific role of this cycle in morphine-induced metabolite changes within the RVM.

The observed increase in GSH and Tau levels following morphine administration in the wild-type mice suggests possible modulation of antioxidant-related pathways. GSH is a major intracellular antioxidant that protects against reactive oxygen species (ROS), while Tau functions in osmoregulation and also exhibits neuroprotective and antioxidant properties [27,28]. Previous studies have shown that chronic morphine exposure leads to elevated oxidative stress, which can upregulate the synthesis of endogenous antioxidants such as GSH and Tau [29–32]. In our study, these metabolite changes may reflect an adaptive neurochemical response to morphine rather than direct evidence of oxidative stress, as ^1^H MRS does not measure oxidative stress. In contrast, the absence of significant changes in GSH and Tau levels in GIRK2^+^/^−^ mutant mice suggests that GIRK2 channel dysfunction may alter morphine-induced modulation of antioxidant metabolites. These findings raise the possibility that the GIRK2 channel mutation has impacted opioid-induced oxidative stress responses.

*N*-acetylaspartate (NAA), a well-established marker of neuronal viability and energy metabolism [33–35], exhibited differential responses to morphine in wild-type versus the GIRK2+/− mutant mice. In wild-type mice, NAA concentrations significantly decreased following morphine administration, whereas no such change was observed in the GIRK2+/− mutants. These findings in wild-type mice align with previous animal studies that have documented morphine-induced reductions in NAA across various brain regions, including the nucleus accumbens and medial prefrontal cortex [15,16]. NAA is frequently used as a diagnostic biomarker for several central nervous system (CNS) disorders, with consistent reductions reported in conditions such as brain ischemia, multiple sclerosis and Alzheimer’s disease [36–38]. Its concentrations are positively correlated with other markers of neuronal metabolism in both animal and human studies [39–41], and declines in NAA typically reflect neuronal damage, loss, or reduced metabolic activity [41–43]. In the present study, the observed decrease in NAA in wild-type mice likely reflects morphine-induced alterations in neuronal metabolism. The absence of this metabolic shift in the GIRK2+/− mice suggests that GIRK2 channels contribute to morphine’s effects on neuronal metabolic activity.

Both the wild-type and the GIRK2 mutant mice exhibited decreased NAA + NAAG levels following morphine administration. In wild-type animals, this reduction was accompanied by a drop in NAA. This complicates the interpretation due to the spectral overlap between NAA and NAAG in ^1^H NMR spectroscopy. NAAG acts as an inhibitory neurotransmitter; it activates presynaptic mGluR3 receptors, suppresses glutamate release, and provides neuroprotection against excitotoxicity [44]. The simultaneous decrease in NAA and NAA + NAAG in the wild-type mice, therefore, makes it impossible to determine whether NAAG levels alone declined. However, in GIRK2 mutants, the decrease in NAA + NAAG occurred without a change in NAA, suggesting a selective reduction in NAAG. Whether this reflects morphine-induced dysregulation of glutamatergic signaling via NAAG/mGluR3 pathways or other neuronal activity changes in the RVM requires further investigation.

The combined choline-containing metabolites GPC + PCh increased in the RVM of wild-type mice but decreased in GIRK2^+^/^−^ mutants following morphine exposure. GPC + PCh are markers of membrane phospholipid turnover and integrity, reflecting the balance between synthesis and degradation of membrane components [45]. The increase observed in wild-type mice likely represents adaptive membrane remodeling in response to morphine-induced metabolic or oxidative stress. In contrast, the decrease in GPC + PCh in GIRK2^+^/^−^ mice suggests that GIRK2 dysfunction may impair the membrane-related metabolic responses normally engaged during sustained opioid exposure.”

### 4.1. Limitations

While this study provides valuable insights into metabolite alterations in the RVM following morphine administration, several limitations should be noted. To improve the low signal-to-noise ratio (SNR) in small-animal NMR spectroscopy, a relatively large voxel size was used. Although this enhances the reliability of metabolite detection particularly in deep brain structures like the RVM, it increases partial volume effects and may introduce signals from adjacent regions, reducing anatomical specificity. Moreover, ^1^H NMR spectroscopy lacks cell-type resolution, limiting our ability to attribute observed metabolic changes to specific cell populations such as neurons or glia. Additionally, placebo pellet implantation itself induced metabolite changes in the RVM. Such changes were likely due to surgical stress, local inflammation, or tissue responses [46]. However, these nonspecific effects were expected to be minimal in the morphine group due to the well-documented analgesic and anti-stress properties of opioids [47,48]. Although both male and female animals were used in the present study, sex was not used as a biological variable in the data analysis, thereby limiting the ability to assess potential sex-specific differences in opioid responsiveness, including GIRK-mediated mechanisms. Another limitation of this study is that metabolite values were summarized as weighted group means, so variability among individual mice could not be directly measured. Since we did not apply family-wise corrections such as Bonferroni, some inflation of Type I error is possible; therefore, results should be interpreted as coherent metabolic patterns rather than solely by individual *p*-values. Inter-session (test–retest) reproducibility within the same animals was not assessed; however, low intra-scan and inter-subject variability, together with consistent treatment and genotype-specific metabolite changes, suggest that the observed longitudinal effects reflect biological, rather than measurement, variability. Lastly, this study did not include behavioral testing to confirm the presence or absence of analgesia or hyperalgesia related to morphine-induced changes in the RVM metabolites. Future studies should therefore incorporate quantitative behavioral assays to correlate metabolite profiles with nociceptive sensitivity.

### 4.2. Conclusion

The results of the present ^1^H NMR spectroscopy study demonstrate that the GIRK2^+/−^ mutation substantially alters the morphine-induced metabolite changes in the RVM. In the wild-type mice, these changes reflected decreases in energy metabolism, increases in antioxidant defense, and disturbances in the Glu + Gln cycle. In GIRK2^+/−^ mice, these responses were either absent or differed in magnitude and/or direction of change compared to the wild-type mice. These results likely represent widespread neurochemical adaptations in the RVM following sustained morphine exposure, rather than changes restricted to a particular functional pathway. The observed metabolite changes were supported by low intra-scan and inter-subject variability, and while inter-session reproducibility was not assessed, the data suggest that these effects primarily reflect biological, rather than measurement, variability. These findings nonetheless indicate that GIRK2 channel function is necessary for the full expression of morphine-induced metabolic changes, highlighting its role in shaping RVM neurochemistry in response to sustained opioid exposure.

## Supplementary Material

MMC1

MMC2

MMC3

## Data Availability

Data will be made available on request.

## Appendix A. Supplementary data

Supplementary data to this article can be found online at https://doi.org/10.1016/j.mri.2026.110668.
