# Supplementary material for: Role of GIRK2 channels in morphine-induced metabolite changes in the rostral ventromedial medulla
Source: Magn Reson Imaging. Author manuscript; Available in PMC 2026 Jun 25. (PMC13295623; doi:10.1016/j.mri.2026.110668)
Supplement: MMC1 [file NIHMS2161442-supplement-MMC1.docx]

**Supplementary Table 1**

| **Metabolite** | **Placebo Day 0** | | **Placebo Day 4** | | **Morphine Day 0** | | **Morphine Day 5** | |
| --- | --- | --- | --- | --- | --- | --- | --- | --- |
|  | **Mean ± SD** | **%SD** | **Mean ± SD** | **%SD** | **Mean ± SD** | **%SD** | **Mean ± SD** | **%SD** |
| PCr | 4602.33 ± 171.58 | 3.73 | 4046.62 ± 204.79 | 5.06 | 4233.18 ± 189.47 | 4.48 | 4600.67 ± 211.43 | 4.60 |
| Gln | 1353.70 ± 200.64 | 14.82 | 1291.93 ± 215.59 | 16.69 | 1079.72 ± 182.58 | 16.91 | 1533.25 ± 226.99 | 14.80 |
| Glu | 4210.84 ± 226.62 | 5.38 | 3891.36 ± 240.53 | 6.18 | 3984.36 ± 212.10 | 5.32 | 4221.07 ± 243.96 | 5.78 |
| Gsh | 638.76 ± 98.51 | 15.42 | 772.20 ± 105.76 | 13.70 | 640.12 ± 93.86 | 14.66 | 839.03 ± 112.65 | 13.43 |
| Ins | 4928.16 ± 167.43 | 3.40 | 5004.15 ± 181.13 | 3.62 | 4759.58 ± 155.60 | 3.27 | 4834.26 ± 184.87 | 3.82 |
| NAA | 4537.24 ± 147.79 | 3.26 | 4203.16 ± 166.37 | 3.96 | 4506.46 ± 148.36 | 3.29 | 4002.70 ± 167.68 | 4.19 |
| Tau | 936.19 ± 167.95 | 17.94 | 1260.05 ± 197.46 | 15.67 | 936.29 ± 162.14 | 17.32 | 1311.43 ± 213.82 | 16.30 |
| GPC+PCh | 823.65 ± 40.25 | 4.89 | 810.41 ± 43.99 | 5.43 | 801.06 ± 38.64 | 4.82 | 856.78 ± 45.79 | 5.35 |
| NAA+NAAG | 5543.83 ± 145.15 | 2.62 | 5239.81 ± 156.11 | 2.98 | 5574.24 ± 142.80 | 2.56 | 5224.42 ± 167.36 | 3.20 |
| Cr+PCr | 5021.65 ± 128.94 | 2.57 | 4948.71 ± 137.93 | 2.79 | 4653.10 ± 116.58 | 2.51 | 4986.97 ± 146.61 | 2.94 |
| Glu+Gln | 5651.18 ± 266.29 | 4.71 | 5195.73 ± 288.24 | 5.55 | 5124.73 ± 244.15 | 4.76 | 5776.37 ± 298.03 | 5.16 |

**Table 1.** Concentrations of metabolites in the rostral ventromedial medulla (RVM) of Wild-type mice on Day 0 and Day 4 after implantation with placebo or morphine pellets. Values are mean ± SD, with the adjacent %SD indicating the LCModel Cramér–Rao lower bound (CRLB) for each metabolite estimate.
