# Supplementary material for: Role of GIRK2 channels in morphine-induced metabolite changes in the rostral ventromedial medulla
Source: Magn Reson Imaging. Author manuscript; Available in PMC 2026 Jun 25. (PMC13295623; doi:10.1016/j.mri.2026.110668)
Supplement: MMC2 [file NIHMS2161442-supplement-MMC2.docx]

**Supplementary Table 2**

| **Metabolite** | **Placebo Day 0** | | **Placebo Day 4** | | **Morphine Day 0** | | **Morphine Day 4** | |
| --- | --- | --- | --- | --- | --- | --- | --- | --- |
|  | **Mean ± SD** | **%SD** | **Mean ± SD** | **%SD** | **Mean ± SD** | **%SD** | **Mean ± SD** | **%SD** |
| PCr | 3066.29 ± 204.27 | 6.66 | 4811.14 ± 181.97 | 3.78 | 4365.08 ± 150.12 | 3.44 | 4496.28 ± 202.51 | 4.50 |
| Gln | 1253.69 ± 179.49 | 14.32 | 1647.76 ± 203.40 | 12.34 | 1728.31 ± 176.60 | 10.22 | 1385.46 ± 203.77 | 14.71 |
| Glu | 3895.56 ± 198.88 | 5.11 | 4118.25 ± 211.71 | 5.14 | 3701.47 ± 189.15 | 5.11 | 4179.11 ± 223.96 | 5.36 |
| Gsh | 678.35 ± 85.73 | 12.64 | 813.39 ± 102.08 | 12.55 | 836.83 ± 88.15 | 10.53 | 869.93 ± 99.27 | 11.41 |
| Ins | 4567.77 ± 149.72 | 3.28 | 4717.15 ± 153.52 | 3.25 | 5033.35 ± 140.98 | 2.80 | 5121.49 ± 167.97 | 3.28 |
| NAA | 4480.42 ± 126.67 | 2.83 | 4742.48 ± 148.42 | 3.13 | 4603.28 ± 125.97 | 2.74 | 4513.13 ± 143.09 | 3.17 |
| Tau | 1366.58 ± 148.22 | 10.85 | 1306.19 ± 172.84 | 13.23 | 1317.92 ± 141.75 | 10.76 | 1269.55 ± 186.79 | 14.71 |
| GPC+PCh | 736.78 ± 35.67 | 4.84 | 717.00 ± 38.00 | 5.30 | 902.33 ± 34.16 | 3.79 | 860.90 ± 40.92 | 4.75 |
| NAA+NAAG | 5741.39 ± 131.15 | 2.28 | 5611.81 ± 136.34 | 2.43 | 5724.95 ± 122.01 | 2.13 | 5467.19 ± 141.40 | 2.59 |
| Cr+PCr | 4951.23 ± 114.86 | 2.32 | 4947.75 ± 118.23 | 2.39 | 5018.91 ± 107.07 | 2.13 | 4879.79 ± 128.55 | 2.63 |
| Glu+Gln | 5197.89 ± 239.76 | 4.61 | 5621.98 ± 251.43 | 4.47 | 5456.59 ± 226.63 | 4.15 | 5612.17 ± 265.63 | 4.73 |

**Table 2.** Concentrations of metabolites in the rostral ventromedial medulla (RVM) of GIRK2⁺/⁻ mice on Day 0 and Day 4 after implantation with placebo or morphine pellets. Values are mean ± SD, with the adjacent %SD indicating the LCModel Cramér–Rao lower bound (CRLB) for each metabolite estimate.
