# Supplementary material for: Role of GIRK2 channels in morphine-induced metabolite changes in the rostral ventromedial medulla
Source: Magn Reson Imaging. Author manuscript; Available in PMC 2026 Jun 25. (PMC13295623; doi:10.1016/j.mri.2026.110668)
Supplement: MMC3 [file NIHMS2161442-supplement-MMC3.docx]

**Table 3 – Reproducibility Data**

|  | NAA+NAAG | | GPC+PCh | | Cr+PCr | | Glu+Gln | |
| --- | --- | --- | --- | --- | --- | --- | --- | --- |
|  | WT | GIRK2+/- | WT | GIRK2+/- | WT | GIRK2+/- | WT | GIRK2+/- |
| M | 5607.86 | 5595.63 | 813.34 | 810.46 | 4922.86 | 4808.13 | 5665 | 5383.13 |
| SD | 554.24 | 560.74 | 169.49 | 125.57 | 670.88 | 595.0487 | 434.1526 | 899.29 |
| CV | 9.88% | 10.02% | 20.84% | 15.49% | 13.63% | 12.38% | 7.66% | 16.71% |
| Min | 4680 | 4790 | 523.87 | 638.59 | 3850 | 3280 | 4970 | 3990 |
| Max | 6530 | 6370 | 1200 | 1100 | 6150 | 5610 | 6380 | 7030 |
| MSD% | 9.29 | 7.56 | 19.86 | 15.19 | 9.50 | 7.50 | 16.14 | 14.25 |

Wild Type (WT) n=14, GIRK2+/- n=16

M = Mean
SD = Standard Deviation
CV = Coefficient of variability in %
MSD% = Mean SD%

**Table 4– Combined Day 0 Reproducibility Data**

|  | NAA+NAAG | GPC+PCh | Cr+PCr | Glu+Gln |
| --- | --- | --- | --- | --- |
| M | 811.81 | 5601.33 | 4861.67 | 5514.67 |
| SD | 145.04 | 548.07 | 623.13 | 723.37 |
| CV | 17.87% | 9.78% | 12.82% | 13.12% |
| Min | 523.873 | 4680 | 3280 | 3990 |
| Max | 1200 | 6530 | 6150 | 7030 |
| MSD% | 17.37 | 8.37 | 8.43 | 15.13 |

Combined n=30

M = Mean
SD = Standard Deviation
CV = Coefficient of variability in %
MSD% = Mean SD%
